# Beyond the ICU: family resilience and emotional turmoil after intensive care – a qualitative study

**DOI:** 10.1080/17482631.2026.2647085

**Published:** 2026-03-21

**Authors:** Jenny Lönnkvist, Tina Lundberg, Eva Åkerman, Ann-Charlotte Falk, Lena Anmyr, Oili Dahl

**Affiliations:** a Department of Clinical Science, Intervention and Technology (CLINTEC), Karolinska Institute, ANA FUTURA, Huddinge, Sweden; b Function Perioperative Medicine and Intensive Care, Karolinska University Hospital, Huddinge, Sweden; c The Deparment of Social Work, Marie Cederschiöld University, Stockholm, Sweden; d Department of Women's Health and Allied Health Professionals Theme, Karolinska University Hospital, Stockholm, Sweden; e Department of Health Sciences, Lund University, Lund, Sweden; f Swedish Society in Nursing, Stockholm, Sweden; g Sophiahemmet University, Stockholm, Sweden; h Women’s Health and Allied Health Professionals Theme, Department of Health professionals, Social Work in Health, Karolinska University Hospital, Stockholm, Sweden; i Division of Nursing, Department of Neurobiology, Care Sciences and Society, Karolinska Institute, Stockholm, Sweden

**Keywords:** Communication, family members, family-centred care, family support, intensive care

## Abstract

**Background:**

During the COVID-19 pandemic, visitation restrictions in ICUs intensified psychological distress among family members, yet the long-term impact of psychosocial support on family resilience remains poorly understood.

**Objective:**

To evaluate the experiences of family members during the COVID-19 pandemic, focusing on interactions with healthcare professionals and well-being 18th months after hospitalisation.

**Methods:**

A qualitative descriptive study using semi-structured interviews with 14 families of patients admitted to the ICU in spring 2020.Data were analysed using inductive content analysis.

**Results:**

Experiences were shaped by caring interactions with healthcare professionals, emotional disruption, and reliance on family support. Clear information and psychosocial support fostered trust but were also associated with stress and feelings of isolation. The experience caused profound emotional distress, with family members reporting lasting anxiety, while others described gratitude and personal growth. Support from Family were central to coping, although the responsibility as the primary contact person could increase emotional burden.

**Conclusions:**

Families experienced significant psychological distress and long-lasting effects. The findings underline the need for improved communication, proactive support, and structured family-centred practices to address both immediate and long-term needs. Healthcare systems should implement strategies such as clear communication plans, follow-up counselling, support groups to reduce families’ emotional burden and improve well-being.

## Introduction

The COVID-19 pandemic had a profound global impact, resulting in millions of confirmed cases and deaths. Even in high-income countries like Sweden, healthcare resources in affected areas were quickly overwhelmed, particularly during the first wave of the pandemic. Intensive care units (ICU) were at the forefront of treating the most critically ill patients (Welfare NBoHa, 2023). During the peak in March 2020, approximately 750 patients required ICU care in Sweden, with three out of four being COVID-19 cases, which was more than twice as many intensive care patients as before the pandemic (Welfare NBoHa, 2023). The Stockholm and Sörmland regions were the hardest-hit areas in Scandinavia (Escher et al., 2023).

A systematic review revealed widespread psychological distress among COVID-19 patients (Zhang et al., 2022), placing a significant emotional burden on their families. Family members often experienced heightened stress, anxiety, and depression due to their loved one’s illness (Gayatri & Irawaty, 2022; Scott et al., 2019). During the ICU stay, they commonly reported feelings of fear, detachment, loneliness, disorientation, guilt, and a sense of life being on hold (Bartoli et al., 2021). Maintaining family connections, managing conflicts, and spending quality time together were key factors in promoting well-being during the pandemic (Gayatri & Puspitasari, 2022). Consistent communication between healthcare professionals and families played a crucial role in alleviating distress and reducing negative psychological effects (Bartoli et al., 2021). However, strict visitation policies and the use of personal protective equipment (PPE) created significant barriers to traditional to communication and the involvement of family members (Fernández-Martínez et al., 2022). In response, innovative approaches such as telecommunication proved effective in preserving family involvement and connection (Fernández-Martínez et al., 2022).

Family resilience—commonly understood as the family’s capacity to adapt, reorganise, and find meaning in the face of crisis and prolonged stress (Walsh, 2003)—has been identified as a central factor influencing how families cope with critical illness. Resilience is shaped not only by internal family dynamics but also by the quality of external support, including communication and psychosocial care provided by healthcare professionals.

Recent international research underscores the importance of high-quality nursing care and supportive practice in intensive and other high-acuity settings during crises. A cross-sectional study from West Bank hospitals found significant differences in patient satisfaction between critical care units and medical wards, highlighting the need to strengthen communication and relational care processes (Smerat et al., 2025). Moreover, ICU nurses’ knowledge, attitudes, and self-efficacy regarding care vary considerably, suggesting that staff preparedness influences how psychosocial needs are addressed (Awad et al., 2025). Collectively, these findings indicate that organisational structures, professional competence, and communication practices shape families’ experiences and adaptive capacity.

To support family members during patient isolation and the visitation ban, Karolinska University Hospital established a dedicated helpline—an initiative not previously implemented in Sweden. Operated daily, including weekends, and staffed by medical social workers, the helpline aimed to provide emotional support while also alleviating the burden on ICU staff. As part of the medical unit for social work, medical social workers proactively reached out to family members after patient admission to assess their psychosocial situation, offering crisis support, ongoing contact, and follow-up in the event of a patient’s death.

Investigation of family members' experiences with psychosocial support from healthcare professionals remains limited, particularly regarding the long-term impact of the pandemic on their well-being. Although previous studies have documented acute psychological distress among family members of ICU patients, less is known about how structured communication initiatives and psychosocial support efforts influence families’ longer-term adaptation and resilience. Moreover, few studies have explored these processes within the unique context of prolonged visitation restrictions and large-scale healthcare disruption. Understanding the impact of visitation restrictions and other pandemic-related factors on family resilience is crucial for enhancing support quality and addressing their needs both now and in the future. Addressing this knowledge gap is essential for informing sustainable family-centred support models that remain robust during both crisis situations and routine critical care practice. In light of this context, the study examines family members' experiences of interacting with healthcare professionals and the long-term emotional effects of intensive care during the pandemic.

## Objectives

This study aims to evaluate the experiences of family members patients treated in intensive care units (ICUs) during the COVID-19 pandemic, focusing on their interactions with healthcare professionals as well as their well-being one and a half years after hospitalisation.

## Methods

### Design

This study is part of the larger observational project *Recovery and Rehabilitation during and after COVID-19 (ReCOV)*, a multidisciplinary research programme exploring the experiences of patients, family members, and healthcare professionals during and after COVID-19 care (Lundberg et al., 2024; Rydwik et al., 2021). Within this framework, the present study employed a qualitative descriptive design to explore family members’ experiences of psychosocial support during a relative’s ICU stay.

### Research team and reflexivity

The research team consisted of clinical nurse specialists, medical social workers, and researchers with expertise in intensive care, psychosocial support, and qualitative methods. During the study period, one interviewer (EÅ) was concurrently employed as an intensive care nurse in the ICU. Importantly, none of the patients or family members who participated were personally acquainted with any of the interviewers before data collection commenced. During analysis, reflexive discussions led to deliberate scrutiny of early coding decisions and prompted refinement of categories to ensure that interpretations were grounded in participants’ accounts rather than professional norms. In several instances, preliminary interpretations were revisited and adjusted following team discussions that challenged taken-for-granted clinical perspectives. This ongoing reflexive process strengthened analytical rigour and transparency by making the researchers’ influence on data interpretation explicit and actively managed.

### Sample and setting

Patients who had been admitted to the ICU at a University Hospital in Sweden during the first wave of the COVID-19 pandemic in the spring of 2020 were eligible for inclusion. The University Hospital is a large tertiary hospital with two main sites. Patients who had consented to participate in the ReCOV project (Rydwik et al., 2021) were asked to provide permission for the researchers to contact their family members. Eligibility criteria required that family members be adults, able to communicate in Swedish, and identified as the primary contact person. This designation implied that they were the only individuals authorised to communicate with healthcare professionals throughout the patient’s hospitalisation. A purposeful and convenient sampling strategy was employed. In the autumn of 2021, twenty patients who had previously consented to participate in research were contacted by one of the researchers as part of the recruitment process. These patients were chosen to capture a variation in age, gender and hospital site where they received treatment. Of these, 17 patients authorised contact with their family members, thereby enabling the inclusion of consenting relatives in the study. In this study, the term family members refers to individuals identified by the patients as close family members, regardless of biological or legal relationship, and may include neighbours or friends. The designated family members were contacted by a clinical social worker (EF) or nurse specialist (EÅ/OD) by telephone. Those family members who agreed to participate were provided with written information and subsequently signed a consent form. Of the 17 family members contacted, 15 agreed to participate, while the remaining two were either unreachable or faced language barriers. Later, one of the participants declined to take part, resulting in a total of 14 respondents.

### Data collection

Data were collected between August 2021 and January 2022 through semi-structured, audio-recorded interviews (*n* = 14). The mean interview length was 55 minutes (range 29–95). Thirteen interviews were conducted by video call and one by telephone. Sampling stopped when information power was deemed sufficient, as the dataset provided rich, varied, and detailed accounts addressing the study aim.

#### Development and pilot testing of the interview guide

To ensure methodological consistency across different studies in the ReCOV project, the family interview guide was derived from the patient interview guide. The research team then adapted the guide to align with the study’s objectives and to capture psychosocial support needs from a family perspective. A pilot interview was conducted by an experienced medical social worker, involving a family member who was not included in the main sample. The pilot confirmed that the questions were understandable, relevant, and elicited the intended depth of response; therefore, no revisions were required.

#### Ensuring consistency across interviewers

While four interviewers (EÅ, TL, OD, EF) were involved in conducting the interviews, methodological consistency was safeguarded using a standardised semi-structured interview guide. In addition, the research team engaged in joint training and preparatory discussions, maintained regular check-ins during the interview period, and documented the process systematically.

Each interview began by asking participants to describe their experiences of psychosocial support, communication with healthcare professionals, and their own well-being during and after their relative’s ICU stay. The interview guide included broad, open-ended questions such as: *“How did you experience the care period when your loved one was treated at the hospital?”*, *“How did you perceive the care your loved one received while hospitalised?,”* “*How did you perceive that your loved ones need for emotional support and comfort was met?”* and “*How secure did you feel with the support that was provided to your loved one?”.* These questions served as entry points for further exploration of family members’ perspectives.

### Qualitative data analysis

An inductive qualitative content analysis was conducted according to Graneheim and Lundman (2004). This methodological approach was considered particularly appropriate for investigating complex emotional and psychological experiences. Three authors (JL, TL, OD) independently read and re-read the interview transcripts to obtain a comprehensive understanding of the material. This process preceded the identification of meaning units that were relevant to the study’s research aim.

Meaning units were condensed and coded independently, then compared and refined collaboratively. The development of categories and subcategories was carried out collaboratively during a series of analytical meetings. Continuous discussions within the research team ensured that both manifest content (explicitly stated) and latent content (underlying meanings) were systematically identified and captured. No qualitative software was used; instead, Excel spreadsheets were employed for the systematic organisation of meaning units, codes, and categories. This manual approach was chosen because the dataset was of a manageable size and the analytic process benefited from the researchers’ close, iterative engagement with the material. Using Excel allowed for high transparency in coding decisions and flexible restructuring of categories.

Trustworthiness was strengthened by involving multiple researchers with different professional backgrounds in the analytic process. Reflexive discussions were held continuously to ensure rigour and transparency. Furthermore, an independent audit of the analytic procedure was conducted by another author (AF), and the full research team subsequently reviewed the categories to reach consensus.

### Ethical considerations

All participants received oral and written information and provided written informed consent. They were informed of confidentiality, their right to withdraw at any time, and how their data would be handled.

Given the potentially distressing nature of recalling an ICU experience during the pandemic, participants were offered supportive contact both during recruitment and during or after the interview if needed. Interviewers were clinical professionals experienced in supporting individuals in crisis and were prepared to pause or terminate interviews if distress occurred.

The study followed the Declaration of Helsinki, and the Regional Ethical Review Board confirmed that no separate ethical approval was required for this sub-study within the approved ReCOV project.

## Findings

### Characteristics of the family members

The analysis included 14 family members of patients hospitalised for COVID-19 between 1 April and 12 June 2020. Of these, 10 (71%) were women and 4 (29%) were men. Participants’ ages ranged from 18 to 79 years, with a median age of 50.5 years (interquartile range [IQR] 40–60). Seven participants (50%) were cohabiting with the patient who had been treated in the ICU. The relationships to the patient included spouses (*n* = 6), children (*n* = 3), siblings (*n* = 2), one partner, one friend and one neighbour (Table I).

**Table I.** Overview of the participants' characteristics.

Participant	Age	Sex	Relation	Cohabitating with the patient
1	18	Male	Child	Yes
2	32	Female	Child	No
3	38	Female	Spouse	Yes
4	40	Female	Partner	No
5	44	Female	Sibling	No
6	46	Female	Spouse	Yes
7	47	Female	Child	No
8	54	Male	Spouse	Yes
9	57	Male	Spouse	Yes
10	59	Male	Spouse	Yes
11	60	Female	Spouse	Yes
12	66	Female	Friend	No
13	71	Female	Sibling	No
14	79	Female	Neighbour	No

### The family member’s experiences of interactions and psychosocial support

Three categories with five sub-categories were identified to describe the long-term experiences of family members, both in relation to their contact with healthcare professionals and their own lives, during and after their loved ones’ treatment for COVID-19 in intensive care. The categories—*Caring interaction—key to trust*, *A Disrupted Foundation*, and *Being Rooted in the Family*—illuminate the resilience and adaptability of family members as they navigated the crisis, seeking comfort and understanding in the face of uncertainty; see Table II.

**Table II.** Categories and sub-categories.

Categories	Sub-categories
**Caring interactions - key to trust**	Being reassured by information Support from medical social worker Trust despite doubt
**A disrupted foundation**	Emotional turmoil A new outlook on life
**Being rooted in the family**	

### Caring interactions—key to trust

This category comprises three subcategories: *Being reassured by information*, *Support from a medical social worker*, and *Trust despite doubt*. It captures family members’ experiences of interacting with healthcare professionals, primarily through telephone communication from the ICU. It further explores the emotional support provided by medical social workers via the helpline, as well as the dynamic interplay between trust and uncertainty that shaped their overall experience.

#### Being reassured by information

Family members described relying on telephone updates from healthcare professionals, primarily physicians, in the ICU as a crucial source of information about their loved ones. The family members described the daily calls as a lifeline that provided reassurance amid the uncertainty. However, the experience varied; while some found these updates comforting and informative, others described them as stressful and unclear, struggling to interpret complex medical vocabulary.

The anxiety surrounding these calls often left family members feeling as though they were in a vacuum, neglecting their own needs as they waited anxiously for updates. The fear of missing a call compounded their stress, making it impossible for them to leave their phones unattended. A sister shared:

I slept with the phone more or less in my ear. I hardly dared to do anything. My husband and I thought we'd go out into the woods for a walk, but I didn't dare because the coverage might disappear, I didn't want to be away from the phone for five seconds. (Participant 5)

Some expressed a desire for simpler updates from the healthcare professionals—just knowing their loved one was alive and being cared for would have sufficed for them. Despite the tension, family members valued the honesty and compassion displayed by the professionals, feeling that these calls provided a vital connection and a glimmer of hope during a challenging time.

#### Support from a medical social worker

Navigating a loved one's health crisis was described as emotionally tumultuous, with family members feeling unsupported by healthcare services. Some managed to cope independently:

‘I haven't [received support from the healthcare service], apart from support from my family, no. I have ... sort of built myself up so to speak’ (Participant 1),

while others felt overwhelmed and in need of more support. Family members reported feelings of loneliness and a lack of proactive outreach from healthcare professionals, making it difficult to seek psychosocial support.

#### Trust despite doubt

Despite family members’ descriptions of anxiety and a troubling time, they also expressed a strong sense of trust in the healthcare provided, feeling confident that professionals were doing their utmost for their loved ones. As one husband put it,

‘It felt very safe actually. I was never worried that someone would not take care of her. I trusted them to do exactly what, what was expected ... it felt like they instilled a very good confidence’ (Participant 10).

Gratitude was a common sentiment expressed by family members. Another husband noted,

‘I am still grateful for what the healthcare service did, that they took her seriously … otherwise she would not have survived … It was traumatic, but the lasting feeling is also one of enormous gratitude’ (Participant 9).

However, some family members also faced challenges in their initial interactions with healthcare professionals, describing how they felt their concerns were not always taken seriously.

Family members expressed feeling uninvolved in care decisions, although most indicated that they preferred not to be involved at all.

### A disrupted foundation

This category comprises two subcategories: *Emotional turmoil* and *A new outlook on life*. It explores the profound emotional distress experienced by family members and how these experiences prompted a shift in perspective, cultivating a deeper awareness of life’s fragility and a renewed appreciation of what matters most.

#### Emotional turmoil

Family members described experiencing profound distress during the tumultuous period when their loved one was hospitalised, marked by fear, anxiety, and stress. These feelings were described as enhanced by the fact that COVID-19 was a new disease affecting the community. As one sister expressed,

‘It was still new and that's what was so hard, that nobody really knew … how it would develop, … it was a new disease, and it was very scary’ (Participant 5).

The anxiety was pervasive, reflecting family members’ sense of being overwhelmed by the situation. Another family member emphasised,

‘It felt like a war zone … It was really the panic and the uncertainty, the worst. All that was terrible. And then there was so much media coverage too, you couldn't get a break from it’ (Participant 3).

The constant strain made it challenging to cope with household responsibilities, impacting family dynamics and overall mood. They expressed that the distress became a daily burden, characterised by feelings of chaos and overwhelming worry, leading to sleepless nights and a lack of appetite. The uncertainty surrounding the COVID-19 disease heightening the fears of family members, as both they and, as interpreted by them, healthcare professionals struggled to understand its implications and prognosis.

One of the most difficult aspects for family members was the inability to visit their loved ones in the hospital, leaving them with haunting thoughts of loved ones facing death alone. The situation was described by one wife:

‘It was very unreal, because you never got to go there, I never got to see him sick or on a ventilator or anything like that, so that whole situation was very unreal’ (Participant 6).

This isolation they described was compounding feelings of helplessness and fear. To cope, some engaged in constant activity, finding solace in work and resisting the idleness that came from waiting at home.

There was a divide among the family members: some felt relief as their loved ones began to recover, while others continued to struggle with long-lasting trauma and anxiety, even after their loved ones had returned home.

‘I still get sad when I talk about it. It remains very emotional’ (Participant 6).

They described mixed emotions, where feelings of depression were combined with gratitude, reflecting both sadness and appreciation for the care received. As one daughter expressed it:

‘I’m feeling pretty good overall—a strange mix of feeling down but at the same time full of gratitude for being so privileged. I guess I just have to be content with that’ (Participant 7).

#### A new outlook on life

The COVID-19 disease engaged family members in profound reflections on life and death. They no longer took life for granted, embracing its fleeting nature and valuing even the smallest moments. A wife stated,

‘When you've been through something like this, then all the other everyday trivial things don't seem so damn important’ (Participant 3).

After receiving their loved ones at home, several reported lifestyle changes, becoming more cautious in public and avoiding crowds. Some noticed a change in the demeanour of those around them, finding conversations more reserved. Conversely, a portion of family members felt that life had returned to normal, maintaining their daily routines.

### Being rooted in the family

This category, which has no subcategories, describes the importance of support and comfort from family and friends. Family members emphasised the vital role of communication within their social networks, highlighting how these interactions offered support and understanding, both during the challenging period when their loved one was hospitalised and in the time that followed. As one husband shared,

‘I have very good contact with my brother ... I talk to him about my situation and vice versa ... the little worries I had are calmed by that’ (Participant 8).

Support from partners and the solidarity of friends were described as creating a comforting atmosphere, strengthening bonds, and fostering a deeper sense of closeness.

Family members experienced ambivalence regarding the role of being a designated contact person for communication with the healthcare professionals. While they appreciated receiving firsthand information, communication with the extended family was expressed as becoming an overwhelming burden through constant inquiries and the delivery of difficult news. As one husband expressed:

I had the support of them, but the family was actually, I must admit, a bit difficult in that they were chasing information, they were more worried about my wife than they were about me … They didn't really understand how I was feeling, what I was going through. (Participant 10)

There was a strong desire to share the responsibilities of being a contact person, as doing so could alleviate feelings of isolation and stress, and ease the practical challenges of navigating both healthcare demands and family dynamics.

## Discussion

This study describes the profound emotional and psychological impact on family members of patients critically ill with COVID-19, emphasising how communication, trust, and support shaped their experiences amidst uncertainty and fear. This study adds nuanced insights into how prolonged uncertainty, physical separation, and reliance on remote communication during a pandemic intensified these experiences.

The findings emphasise the critical role of clear, consistent, and comprehensible communication from healthcare professionals. Family members relied on telephone updates, often physician-led, as their primary source of information, which they described as both a lifeline and a source of anxiety. Similar to earlier studies (Forsberg et al., 2024; Jungestrand et al., 2023; Steiner et al., 2024), regular updates were described as reassuring, fostering trust, and reduced feelings of helplessness. However, when information was perceived as insufficient or conveyed in complex medical terminology, it instead contributed to stress and uncertainty, a sentiment described in other studies (Chen et al., 2021; Forsberg et al., 2024; Jungestrand et al., 2023). While physician-led communication ensures medical accuracy, it may not always be optimally accessible to families. In this context, ICU nurses may serve a crucial complementary function. Through their continuous bedside presence and relational engagement, nurses are well positioned to translate medical information into understandable language and provide communication that is both clinically grounded and emotionally attuned (Wubben et al., 2021). Together, these findings emphasise the need for structured and multi-modal communication strategies in the ICU. Scheduling regular updates at fixed times and offering multiple communication channels—such as phone-calls, video-calls, or written summaries—may help enhance clarity and predictability) (Bernild et al., 2021; Chen et al., 2021; Hochendoner et al., 2022; Renckens et al., 2023). Equally important is training healthcare professionals in clear compassionate communication and ensuring that family members are actively invited to ask questions and seek clarification, thereby strengthening their sense of involvement and control (Asadi & Salmani, 2024; Digby et al., 2023; Jungestrand et al., 2023). Future ICU communication models may therefore benefit from formally integrating nurses into structured family communication pathways, positioning their role as a core component of family-centred care rather than relying solely on physician-led updates (Davidson et al., 2017).

Changing practices and introducing new communication structures, a dedicated helpline staffed by medical social-workers, during an ongoing pandemic is challenging. Those who did use the helpline reported mixed experiences—while some found the support helpful, others expressed frustration over unmet expectations, such as the lack of detailed medical updates. Similar challenges were observed in another study (Digby et al., 2023). These findings align with earlier research, highlighting the importance of proactive outreach and tailoring support services to meet the needs of family members (Asadi & Salmani, 2024; Danielis et al., 2022). To enhance the effectiveness of such support systems, healthcare institutions should focus on raising awareness about available services and ensuring that helpline staff are equipped to address both medical and psychosocial concerns (Babac et al., 2018; Lopez-Soto et al., 2021). Proactive outreach—where medical social workers contact family members directly to assess their needs and provide support—could significantly improve satisfaction and reduce feelings of isolation (Babac et al., 2018). These results also illustrate that simply establishing support structures is insufficient; accessibility, clarity of purpose, and alignment with families’ expectations are equally important. Moving forward, these findings support a more systematic integration of patients and family members in care planning and decision-making processes, thereby implementing and strengthening a genuinely family-centred approach to critical care.

One of the main findings of this study is that having a loved one critically ill in the ICU constitutes a profound emotional turmoil for family members, with consequences that extend far beyond the acute phase. During hospitalisation, participants described intense stress, uncertainty, and fear of loss, amplified by visitation restrictions and the unpredictable course of COVID-19. Feelings of unreality, helplessness, and anticipatory grief were common, echoing earlier research on the psychological burden associated with restricted ICU access and prognostic ambiguity (Bartoli et al., 2021; Digby et al., 2023; Forsberg et al., 2024; Laurent et al., 2023; Steiner et al., 2024). Importantly, this emotional turmoil was not resolved with discharge. Months after the ICU stay, many family members continued to report lingering anxiety, hypervigilance, emotional exhaustion, and altered family dynamics. For some, the experience remained intrusive and trauma-like, consistent with evidence that family members of ICU patients are at risk of persistent post-traumatic stress symptoms extending well beyond 18 months (Nosaka et al., 2024; Rai et al., 2025). These findings reinforce that critical illness is not an isolated medical event but a potentially life-altering family crisis with enduring psychological and relational consequences. At the same time, narratives also revealed elements of family resilience. Several participants described strengthened relationships, reprioritised values, and a renewed appreciation of everyday life. Such adaptive shifts suggest that families engage in meaning-making processes alongside distress, highlighting resilience not as the absence of suffering, but as the capacity to endure, adapt, and reorganise in the face of adversity. Clinically, this duality—coexisting vulnerability and resilience—underscores the importance of structured psychosocial follow-up for family members after ICU discharge. Supporting families should therefore extend beyond mitigating distress to actively fostering adaptive coping and resilience as integral components of post-ICU care (Asadi & Salmani, 2024; Danielis et al., 2022).

Family members in this study described the dual-edged nature of serving as the primary contact person for the healthcare team. While this role ensured they received first-hand information, it also became a source of emotional and logistical strain due to the responsibility of relaying updates to extended family and managing their concerns. The need for multiple family members to share communication responsibilities has been identified in previous research (Conte et al., 2023; Kwame & Petrucka, 2021) to alleviate the burden on the designated contact person.

Taken together, these findings highlight the tension between the principles of family-centred care and the practical restrictions imposed during the pandemic. Although family involvement was necessarily limited, the experiences described by participants underscore how essential relational care, shared understanding, and structured support are for both emotional wellbeing and recovery. Rather than viewing family-centred care as incompatible with crisis conditions, our findings suggest that its core components—clear communication, care continuity, and psychosocial support—become even more critical when physical presence is restricted.

Family-centred care is essential not only for supporting the emotional well-being and resilience of family members but also for improving patient outcomes and care quality (Duong et al., 2024; Forsberg et al., 2024; Wang et al., 2023). ICU care should therefore always strive to be family-centred. However, situations such as pandemics make it even more crucial that these structures are robust and well-established. Developing ICU practices that embed family-centred care in routine care may strengthen healthcare systems’ capacity to maintain meaningful family involvement even during periods of restricted visitation. This includes implementing structured communication strategies, ensuring continuity in information sharing, and providing proactive psychosocial support. Prioritising structured family involvement can strengthen trust, reduce distress, and contribute to better recovery for both patients and their families.

### Limitations

This study used a purposeful and convenient sampling strategy where participants were recruited through patients, which may have introduced bias. Although efforts were made to include diverse backgrounds, relying on patient consent could have placed pressure on them to involve family members. Nevertheless, the variation in participants and the achievement of data saturation strengthen the credibility of the findings. Data were collected retrospective, which may have introduced recall bias. Family members’ memories and interpretations could have been influenced by time and subsequent experiences. Furthermore, participants were limited to those designated as the primary contact person during the ICU stay. This excludes perspectives from other family members who may have had different experiences of psychosocial support. Most interviews were carried out through video conferencing, and one by telephone, as necessitated by the pandemic. These formats may have affected both rapport and the richness of responses relative to in-person interviews. During the analysis process, the reflexive analytical approach among researchers helped reduce bias and enhance trustworthiness. By scrutinising early coding and refining categories through team discussions, interpretations remained grounded in participants’ accounts. Revisiting preliminary conclusions and explicitly managing researcher influence increased transparency and strengthened analytical rigour. The study was conducted in two hospitals in Sweden during the first wave of the COVID-19 pandemic. Consequently, the findings may not be transferable to other healthcare systems, cultural contexts, or subsequent phases of the pandemic. In addition, the small sample size could further limit the transferability of the results. Finally, the possibility of exclusion based on language proficiency presents a risk, potentially reducing sample diversity and omitting perspectives from minority language groups.

#### Implications for practice and research

Future guidelines should consider the emotional and psychological needs of families, particularly during times of crisis and restricted visitation. Lessons learned from the COVID-19 pandemic extend beyond that specific context and should inform best practices in critical care. Families of critically ill patients often face similar challenges regardless of diagnosis. Therefore, it is crucial to prioritise key aspects such as the psychological impact of critical illness, the need for clear and compassionate communication, and the provision of structured support within clinical practice.

In practical terms, this could involve implementing structured communication strategies, such as scheduling daily updates at predetermined times and assigning a designated contact person—often an ICU nurse—to ensure continuity and clarity in information sharing. Another example could be the introduction of routine post-ICU follow-up calls or family consultations within the first months after discharge, aimed at identifying ongoing psychological distress and facilitating referral to appropriate psychosocial support services.

Further research is needed to better understand family members’ needs and perspectives, ensuring that they are actively involved in shaping future interventions. While healthcare professionals may perceive communication as adequate, what matters most is how families experience and interpret these interactions. Building on this knowledge, future work should design and evaluate multiprofessional interventions that include structured communication strategies, follow-up counselling, support groups, and educational resources. Such initiatives may help reduce the long-term emotional burden on family members and support sustainable recovery for both patients and their families.

## Conclusion

The experiences of family members underscore the profound emotional challenges of having a loved one critically ill in the ICU. The findings highlight the need for improved communication, proactive support, and the implementation of family-centred care practices that address both immediate and long-term needs. By establishing strategies such as structured communication, follow-up counselling, support groups, and enhanced family involvement in communication and care processes, healthcare systems can better support families and alleviate the emotional burden associated with critical illness. Additionally, it is essential to recognise the importance of long-term support to ensure that family members receive the care and resources necessary to recover and rebuild their lives after such profound experiences.

## Data Availability

The datasets generated and analysed during the current study are not publicly available due to ethical approval and the anonymity of respondents but are available from the corresponding author, OD, on reasonable request.
